# 3D printing and virtual planning in maxillofacial lesion management: A retrospective study

**DOI:** 10.6026/973206300214043

**Published:** 2025-11-15

**Authors:** Astha Doshi, Ashima B Behl, Alefiya Jakiuddin, Madhuriya Lodha, Saloni Verma, Aanchal Gupta

**Affiliations:** 1 Department of Public Health Dentistry, Kanti Devi Dental College and Hospital, Mathura, Uttar Pradesh, India; 2 Department of Oral Medicine and Radiology, Baba Jaswant Singh Dental College and Research Institute, Ludhiana, Punjab, India; 3 Private Practitioner, Udaipur, Rajasthan, India; 4 Department of Oral and Maxillofacial Surgery, Darshan Dental College and Hospital, Udaipur, Rajasthan, India; 5 Department of Oral Pathology and Microbiology, King George's Medical University, Lucknow, Uttar Pradesh, India; 6 Department of Oral Medicine and Radiology, M.M College of Dental Sciences and Research, Mullana, Haryana, India

**Keywords:** 3D printing, surgical guide, oral lesions, maxillofacial surgery, virtual surgical planning, digital workflow

## Abstract

The integration of three-dimensional (3D) printing and virtual planning in oral and maxillofacial surgery has enhanced precision and individualized patient care is of interest. Therefore, it is of interest to evaluate 42 cases of oral and maxillofacial lesions managed with the aid of virtual planning and 3D printed models/guides. Odontogenic cysts were the most common lesions and the mandible was predominantly affected. Use of 3D models reduced operative time, improved resection accuracy and minimized intraoperative complications, with uneventful recovery in most cases. Overall, 3D printing and virtual planning proved to be reliable tools for improving surgical outcomes and clinical efficiency in maxillofacial lesion management.

## Background:

The management of oral and maxillofacial lesions is challenging due to the region's complex anatomy, functional demands and aesthetic considerations [1]. Conventional diagnostic and surgical methods, while effective, primarily rely on two-dimensional imaging and surgeon expertise, which may limit precision in planning and execution [2]. Technological advances have introduced three-dimensional (3D) printing and virtual surgical planning (VSP) as transformative tools in oral and maxillofacial surgery (OMFS) [3]. 3D printing enables the fabrication of patient-specific anatomical models, surgical guides and implants using imaging data from computed tomography (CT) or cone-beam CT (CBCT) [4]. These replicas enhance spatial understanding, support preoperative simulation, improve intraoperative navigation and facilitate postoperative assessment [5]. VSP allows surgeons to digitally simulate resections, design reconstruction strategies and customize fixation techniques, thereby reducing operative time and minimizing intraoperative errors [6]. Its integration with 3D printing has demonstrated clinical benefits, including improved accuracy, reduced morbidity and enhanced patient-specific care [7].

Applications include management of odontogenic tumors, cysts, benign neoplasms, traumatic defects and complex reconstructions using vascularized flaps or custom implants [8]. Despite increasing adoption, clinical evidence remains limited, with relatively few studies documenting real-world outcomes of 3D-assisted approaches in OMFS [9]. Systematic evaluation is required to determine feasibility, impact and limitations in routine practice. Therefore, it is of interest to analyze the application of 3D printing and VSP in managing oral and maxillofacial lesions, focusing on indications, workflow, outcomes and challenges in a tertiary care setting.

## Materials and Methods:

## Study design and setting:

This retrospective descriptive study was conducted in the Department of Oral and Maxillofacial Surgery, Private Dental College and Hospital, a tertiary referral center, between January 2023 and December 2024. Ethical approval was obtained (IRB Protocol No. 14/2023) and all data were anonymized in accordance with the Declaration of Helsinki.

## Inclusion criteria:

[1] Patients diagnosed with oral and maxillofacial lesions (odontogenic tumors, cysts, benign neoplasms, traumatic defects, or congenital deformities).

[2] Underwent surgical management with virtual surgical planning (VSP) and/or three-dimensional (3D) printing.

[3] Complete imaging, surgical and follow-up records for ≥6 months.

## Exclusion criteria:

[1] Cases managed without 3D printing or VSP.

[2] Incomplete documentation.

[3] Malignant lesions requiring oncologic resection with adjuvant therapy.

## Sample selection:

From hospital archives, 42 patients (n = 42 surgical sites) were identified using procedure codes for 3D-assisted surgeries and selected by purposive sampling.

## Data collection:

Parameters recorded included:

[1] Demographics: Age, gender.

[2] Diagnosis: Lesion type and location.

[3] Imaging: CBCT or MDCT.

[4] Planning software:Exoplan®, Mimics®, Materialise®, or Blue Sky Plan®.

[5] 3D printing: Printer type (FDM, SLA, DLP, or SLS), material (PLA, resin, nylon, titanium) and model type (diagnostic model, cutting guide, reconstructive plate, implant template).

[6] Surgical data: Procedure type, intraoperative guidance, operative time, modifications, complications.

[7] Outcomes: Accuracy of fit, functional/aesthetic results, revision need, surgeon satisfaction (Likert scale 1-5).

## Imaging and planning workflow:

High-resolution CBCT or MDCT scans (DICOM format) were segmented in planning software. Depending on surgical objectives, anatomical replicas, cutting guides, or custom implants were virtually designed. Guides were aligned to anatomical landmarks for resection and reconstruction.

## 3D printing:

STL files were exported to printers according to purpose:

[1] Diagnostic models: FDM with PLA.

[2] Surgical guides: SLA/DLP with biocompatible resin.

[3] Custom implants: SLS or outsourced titanium fabrication.

[4] Post-processing included curing, cleaning and sterilization (ethylene oxide or autoclave).

## Surgical protocol:

Surgeries were performed by experienced OMFS surgeons. Sterilized guides/models were used intraoperatively for osteotomy, lesion excision, or flap positioning. Reconstruction employed pre-bent plates, patient-specific implants, or vascularized grafts as required.

## Outcome measures:

[1] Surgical accuracy: Congruence of postoperative results with virtual plan (CBCT overlays, surgeon evaluation).

[2] Operative time: Compared with historical non-3D-assisted cases.

[3] Complications: Guide misfit, intraoperative deviation, infection, or revision need.

[4] Surgeon satisfaction: Likert scale ratings.

## Data analysis:

Data were analyzed using SPSS v25.0 (IBM Corp., Armonk, NY, USA). Descriptive statistics (mean, SD, percentages) were calculated. Operative time and complication rates were compared with non-assisted cases using independent t-tests or Mann-Whitney U tests. A p-value <0.05 was considered significant.

## Results:

Comparative analysis evaluated operative time, accuracy, complications and surgeon satisfaction. One-way ANOVA and Fisher's Exact Test were applied; p < 0.05 was considered significant. As shown in Table 1 (see PDF), the mean operative time differed significantly between groups (F = 14.37, p < 0.001). The 3D-assisted group recorded the shortest time (93.6 ± 15.7 min) compared to the conventional group (115.8 ± 18.4 min). Stratified by lesion type (Table 2 - see PDF), no significant difference was observed (p = 0.284), although odontogenic tumors required the longest durations. Surgical accuracy, assessed on a 5-point Likert scale, is summarized in Table 3 (see PDF). In the 3D-assisted group, 69.0% achieved the maximum score (5); none scored below 3. Accuracy varied by printing technology (Table 4 - see PDF) (F = 3.35, p = 0.048): SLA produced the highest mean score (4.8 ± 0.4), followed by SLS (4.7 ± 0.5) and FDM (4.5 ± 0.6). Postoperative complications occurred in 3 of 42 cases (7.1%) (Table 5 - see PDF): two surgical guide misfits (FDM) and one infection (SLA). No association was found between complication rate and printing technology (p = 0.281). Overall satisfaction is shown in Table 6 (see PDF). Most procedures (81.0%) were rated "extremely useful" (score 5); none were rated unhelpful. Satisfaction correlated positively with case complexity (Table 7 - see PDF) (F = 3.97, p = 0.035), with complex reconstructions achieving the highest score (5.0 ± 0.0). Planning software had no significant effect on operative time (Table 8 - see PDF) (F = 0.32, p = 0.725). Mean times were: Mimics (92.4 ± 14.2 min), Blue Sky Plan (95.6 ± 16.1 min) and Exoplan (91.3 ± 15.7 min). Satisfaction stratified by complexity (Table 9 - see PDF) confirmed increasing appreciation for 3D technology: cyst enucleations (4.6 ± 0.4), tumor resections (4.8 ± 0.3) and complex reconstructions (5.0 ± 0.0). The difference was significant (F = 3.97, p = 0.035).

## Discussion:

The present study demonstrated that the integration of three-dimensional (3D) printing and virtual surgical planning (VSP) substantially enhanced surgical precision, reduced operative duration, and increased surgeon satisfaction in oral and maxillofacial procedures, without elevating complication rates. The 3D-assisted cohort exhibited significantly shorter operative times compared with conventional techniques, corroborating previous findings that patient-specific templates and pre-fabricated guides streamline both resection and reconstruction phases of surgery [7, 10]. Similar outcomes were reported by Zoabi *et al.* (2022) [5], who concluded that digital planning combined with additive manufacturing improved procedural predictability and intraoperative efficiency in complex maxillofacial interventions. Singhal *et al.* (2022) [11] further reinforced these observations, highlighting that computer-assisted design and manufacturing significantly improved the precision of osteotomies and implant placement while minimizing intraoperative uncertainty. The ability to visualize and simulate surgery preoperatively allowed surgeons to focus on precise execution, minimizing intraoperative decision-making and enhancing overall workflow efficiency. The superior alignment scores observed in the 3D-assisted group reaffirmed the clinical value of digital planning tools. Among additive manufacturing modalities, stereolithography (SLA) demonstrated the highest dimensional fidelity, consistent with prior studies reporting that SLA's finer resolution offered improved surface detail and accuracy compared with fused deposition modeling (FDM) or selective laser sintering (SLS) systems [5, 6 and 12]. Li *et al.* (2024) [7] reported similar findings, showing that SLA-produced guides yielded superior adaptation and reduced fitting discrepancies compared with FDM-based alternatives in mandibular reconstruction. Conversely, Shaheen *et al.* (2018) [13] observed minor deviations in FDM-based templates, attributing them to thermoplastic shrinkage and printing resolution limitations. These contrasting findings collectively indicated that the choice of printing modality remained a key determinant of clinical precision, especially in anatomically complex regions. The observed enhancement in surgeon satisfaction, particularly in multi-segmental and anatomically challenging cases, reflected the psychological and technical benefits of 3D planning in complex surgical contexts. This was consistent with Zoabi *et al.* (2022) [5], who reported that 3D-VSP exerted its greatest clinical impact in reconstructive cases involving extensive bone defects, angular deformities, or customized implants. Bhatt *et al.* (2025) [14] similarly concluded that the integration of VSP improved surgeon confidence, spatial awareness, and intraoperative decision-making during segmental mandibular reconstructions. However, Louvrier *et al.* (2017) [15] reported that in simpler, single-segment osteotomies, digital assistance did not significantly influence operative time or clinical outcome, suggesting that the benefit of 3D workflows may be proportional to procedural complexity. This nuanced finding underscored that while digital planning was universally beneficial for precision, its impact on efficiency may have varied depending on surgical difficulty. Interestingly, this study found that the choice of planning software did not significantly influence operative efficiency or outcomes. This observation agreed with Brandt *et al.* (2015) [16], who suggested that surgeon expertise, rather than the specific software platform, was the dominant factor influencing surgical accuracy. Most VSP systems now provide standardized core functionalities-such as segmentation, virtual osteotomy, and guide design-rendering the surgeon's adaptability and experience more decisive than the digital interface itself. Hence, the integration of digital workflows should prioritize comprehensive user training and interdisciplinary collaboration over software preference. Importantly, no significant increase in complication rates was observed in the 3D-assisted group, supporting the consensus that digital planning enhanced safety and reproducibility without adding procedural risk. Similar observations were made by Ostas *et al.* (2022) [17] and Nesic *et al.* (2020) [18], who reported that postoperative complications, implant misfit, or flap failure rates were comparable between digitally planned and conventionally performed reconstructions. In contrast, Tunchel *et al.* (2016) [19] noted that errors in guide positioning or resin deformation during sterilization could lead to minor intraoperative discrepancies, emphasizing the need for standardized validation and sterilization protocols. The minimal intraoperative issues encountered in the present study, which were readily managed without secondary intervention, supported the high clinical reliability of the workflow employed. The overall acceptance of the technology among surgeons was notably high, with 81% rating it as "extremely useful." This finding reinforced earlier reports suggesting that digital rehearsal and preoperative visualization not only enhanced surgical accuracy but also reduced cognitive load and stress during complex procedures. The psychological preparedness and confidence derived from preoperative simulation have been shown to translate into smoother intraoperative execution and improved postoperative outcomes [20, 21]. In synthesis, the collective evidence - including the present findings and prior literature such as Zoabi *et al.* (2022) [5], Ostas *et al.* (2022) [17], and Bhatt *et al.* (2025) [14] - indicated that the integration of 3D printing and VSP had transitioned from an adjunctive innovation to a cornerstone of modern maxillofacial surgery. While supportive studies consistently emphasized improved accuracy, reduced operative time, and enhanced satisfaction, contrasting evidence highlighted that these benefits were most pronounced in high-complexity cases and depended heavily on surgeon experience and manufacturing precision. Future research should therefore focus on standardizing VSP protocols, evaluating cost-effectiveness, and integrating artificial intelligence and augmented reality into digital workflows to further refine predictive modeling and intraoperative navigation.

## Limitations:

The retrospective design, single-center scope and lack of long-term functional and aesthetic outcomes limit generalizability. Future multicenter, prospective studies should incorporate cost-effectiveness and extended follow-up.

## Conclusion:

3D printing and virtual surgical planning improve the management of oral and maxillofacial lesions by reducing operative time, enhancing accuracy and increasing surgeon satisfaction, particularly in complex cases is shown. Complication rates were low and unaffected by technology type, although SLA provided the highest accuracy. Software choice had minimal impact on efficiency, highlighting the importance of surgical expertise. Thus, we show routine integration of 3D-assisted workflows in preoperative planning, especially for complex reconstructions. Prospective, multicenter studies with long-term outcome analysis are warranted to further validate and optimize clinical utility.
